# COVID-19-related knowledge influences mental health, self-care behaviors, and quality of life among elderly with non-communicable diseases in Northern Thailand

**DOI:** 10.3389/fpubh.2022.993531

**Published:** 2022-10-28

**Authors:** Pattareeya Napalai, Katekaew Seangpraw, Sorawit Boonyathee, Parichat Ong-artborirak

**Affiliations:** ^1^School of Medicine, University of Phayao, Phayao, Thailand; ^2^School of Public Health, University of Phayao, Phayao, Thailand; ^3^Faculty of Public Health, Chiang Mai University, Chiang Mai, Thailand

**Keywords:** COVID-19, mental health, behaviors, quality of life, elderly, NCD

## Abstract

**Background:**

A growing body of research shows that individuals with non-communicable diseases (NCDs), such as hypertension, diabetes, hypercholesterolemia, and heart disease, are more likely to suffer from severe COVID-19 and, subsequently, death. The purpose of this study was to assess the influence of COVID-19-related knowledge on mental health, healthcare behaviors, and quality of life among the elderly with NCDs in Northern Thailand.

**Methods:**

In this cross-sectional study, the participants were 450 elderly people with NCDs, living in the Chiang Rai province, Northern Thailand. Random sampling was applied to select the subjects. Data collection included demographic information, COVID-19-related knowledge, healthcare behaviors, the Suanprung Stress Test-20, the Thai General Health Questionnaire (GHQ-28) for the assessment of mental health, and the Thai version of the World Health Organization Quality of Life-BREF.

**Results:**

Almost half of the participants (45.6%) had poor knowledge about COVID-19. More than half of the sample had high stress (52.0%) and a low score in healthcare behaviors (64.9%), while approximately one-third of the participants had mental health problems (34.0%). The overall quality of life during the COVID-19 pandemic was moderate (70.7%). The score of COVID-19-related knowledge was significantly correlated with scores of stress (*r* = −0.85), mental health (*r* = −0.74), healthcare behaviors (*r* = 0.50), and quality of life (*r* = 0.33). Multiple linear regression found that history of COVID-19 detection and COVID-19-related knowledge were associated with scores of stress and quality of life (*p* < 0.05). Multiple logistic regression showed that history of COVID-19 detection (OR = 4.48, 95% CI = 1.45–13.84) and COVID-19-related knowledge (OR = 0.23, 95% CI = 0.17–0.31) were associated with mental health problem (*p* < 0.05).

**Discussion:**

The findings emphasize the importance of COVID-19-related knowledge concerning the improvement of self-care behaviors and quality of life in the elderly population with NCDs during the pandemic, especially due to the high rate of stress and mental health problems documented in our sample. Health education interventions for this vulnerable population should be organized.

## Introduction

While the world is still in the grip of Coronavirus disease (COVID-19), crucial efforts have been developed to promote learning from the pandemic response (1). The emergence of COVID-19 has had devastating impacts on the healthcare systems around the world and has become a global public health emergency (1). A large body of evidence has shown that severe morbidity and mortality are linked to COVID-19 increases with age (2). The elderly population is one of the groups most vulnerable to COVID-19 infection (3). The worldwide mortality rate from COVID-19 varies across the globe, with some representative examples being the following: 4.1% in China, 4.6% in Spain, and 2.73% in Italy (1, 4). The COVID-19 mortality rate is the highest among individuals aged 80 or more, ranging from 13 to 16.7%, followed by a rate of 7.2–8.9% among people aged 70–79 (4). If infected, people living with non-communicable diseases (NCDs) are at higher risk than those without NCDs of developing severe COVID-19-related illness and death (5). The COVID-19 mortality rate among elderly people induced changes in this population's daily life and increased the fears concerning COVID-19, causing mental health issues such as stress, anxiety, abnormal sleeping patterns, and depression (6, 7). Previous research has found that fear of COVID-19 and poor economic conditions are predictive factors for quality of life among NCDs (8).

In Thailand, the COVID-19 death rate was high among elderly people with patients with NCDs, such as heart diseases and diabetes with severe symptoms (6). According to the Department of Disease Control of the Ministry of Public Health of Thailand, as of May 14, 2022, 4,373,846 cases of COVID-19 were documented, with a total of 29,472 deaths (mortality rate: 0.67%) (9). When classifying the cumulative number of infections among the elderly Thais, 527,878 cases and 21,043 deaths (71.4%) were recorded, with this age group presenting the highest number of deaths within the general population (9). The Chiang Rai province is located in northern Thailand and has the 11^th^ largest population of elderly in the country (237,979; 18.38%) (10). The group of elderly people has the highest mortality rate, with the cumulative number of infections reaching 1,243 (11.56%) and 114 deaths (56.16%) as of May 15, 2022 (6). In addition, the majority of the elderly people infected with COVID-19 have chronic NCDs such as diabetes, high blood pressure, obesity, chronic obstructive pulmonary disease, ischemic heart disease, stroke, and cancer (11).

NCDs represent a major health care challenge, especially in the case of COVID-19 which preferentially impacts people with underlying diseases (12). Patients with specific chronic diseases, such as diabetes, high blood pressure, cardiovascular diseases, and chronic kidney, and liver disease, are more likely to be affected by COVID-19 (9). In this context, people with NCDs infected with COVID-19 are more likely to present poor clinical outcomes than general patients (13). However, patients with NCDs who are infected with COVID-19 were more likely to develop severe illnesses and subsequently die (13). Many health reports suggest that elderly with COVID-19 present serious health complications, especially those with pre-existing diseases, leading to exacerbation of their physical condition and social isolation (7, 14). Consequently, the prevalence and severity of anxiety, stress, and poor mental health related to COVID-19 among the elderly population have increased exponentially (7). Good perception of health, health knowledge, and healthcare behaviors are important for the aging population, especially during the pandemic, since they help increase health awareness (15, 16). Research on the impact of COVID-19 on elderly people with chronic communicable diseases living in rural areas is limited. Therefore, this study aimed to assess the influence of COVID-19-related knowledge on mental health, self-care behaviors, and quality of life among elderly with NCDs living in rural areas of northern Thailand.

## Methodology

### Participants and sampling

The data of this cross-sectional study were collected from October to November 2021, in the Janjawa sub-district of the Chiang Rai Province. This community is located ~30 kilometers from the nearest major urban center, where primary and secondary healthcare systems can be accessed. The population is a diverse mosaic caused by migration. Purposive sampling was employed using the following criteria: (1) provinces located within Health Zone 1; (2) communities with a large number of elderly people, prone to NCDs including hypertension, diabetes mellitus, chronic kidney disease, and stroke; (3) areas affected by the COVID-19 pandemic, where the group of elderly people with health problems further impacted by COVID-19; and (4) local government administrators willing to cooperate with the research team.

Random sampling was employed to select the participants from the list of elderly patients with chronic NCDs registered in the sub-district of Health Promoting Hospital and the community health center program (Java Health Center Information System) during the years 2020–2022. The sample size was calculated using the unknown sample size formula (17), applying a 95% confidence interval (CI) and 5% error level. An additional 10% increase in the sample size led to the recruitment of 450 patients. The inclusion criteria were the following: (1) elderly females and males aged above 60; (2) residents of the research area for at least 1 year, registered in the municipal catalogs; (3) being diagnosed with chronic NCDs (e.g., high blood pressure, diabetes mellitus, heart diseases, and chronic kidney disease) by a medical doctor and receiving health services in the Java Health Center Information System program from 2018 to 2020; (4) being able to communicate in a local language; and (5) signing a written consent form to voluntarily participate in the research. Patients with cognitive and psychological disorders or gestational diabetes were excluded from the study. Five research assistants able to communicate in the local northern language, who served as public health scholars and village health volunteers, were recruited after a public call. A research meeting was organized to clarify the purposes of the study, the data collection technique, the interview procedures, the administration of the questionnaires, the schedule of the interviews, and the rights and privacy of the research participants. The researcher ensured that everyone understood the procedures in the same direction. The questions and interview details were translated from the official language to the local northern language for the optimal understanding of the research questions. An official request was sent to the local Public Health Office asking for permission to conduct the study that was performed during the third wave of COVID-19 in Thailand, requiring authorization by the local government agency and the village headman. All participants complied with the government prevention measures against COVID-19. The data were collected at the local care facility (sub-district Health Promoting Hospital) on the day of clinic visits for patients with chronic NCDs (Monday-Wednesday-Friday) from 9:00 a.m. to 5:00 p.m. or at any other time convenient for the patients. The questionnaires were provided during a presential interview that lasted ~30–45 min per person. A small token of appreciation was given to the participants after completing the survey.

### Measurements

The first part of the questionnaire included the following socio-demographic information: general information (gender, age, marital status, education, occupation, income) and health status (NCD-related health complications, smoking status, alcohol consumption, medication, history of COVID-19, health care services, caregiver, and health information). For the 2–5 questionnaires, the questions were adapted to be suitable for elderly populations of rural contexts based on previous research. As for knowledge related to COVID-19, the questionnaire included 12 items (e.g., “COVID-19 can cause death”, “all patients infected with COVID-19 will have a fever”, etc.) (9, 18). Only one of the three possible answers, “Yes”, “No”, and “Unknown”, could be chosen. The score was 1 point for each correct answer and 0 points for each wrong answer or the “Unknown” option. The scores were divided into three categories: a score range of 80–100% was considered “good knowledge”, 60–79% indicated “moderate knowledge”, and a score range of 0–59% percent was considered “poor knowledge”.

Stress was evaluated with the Suanprung Stress Test questionnaire which consists of 20 items (19). The items assess events that occurred during the past 6 months. The scores were measured on a 5-point scale (1: “no stress”; 2: “mild stress”; 3: “moderate stress”; 4: “a lot of stress”; 5: “extreme stress”). The total scores were used to define four categories: low stress (0–24), moderate stress (25–42), high stress (43–62), and severe stress (equal to or above 63).

The Thai version of the General Health Questionnaire (GHQ-28) consists of 28 items (20) that assess the inability to live in normal conditions and distress-causing problems deriving from a personal abnormal condition (21). The questionnaire does not determine a specific psychiatric disorder (21). Four components are measured using seven items per component: somatic symptoms, anxiety and insomnia, social dysfunction, and severe depression. The participants answered the questions regarding their feelings toward the given statements (both positive and negative) on a 4-point scale, where 1 corresponded to “It doesn't represent me at all”, 2 corresponded to “It represents me/happens to me sometimes”, 3 corresponded to “It represents me/happens to me often”, and 4 corresponded to “It happens to/represents me very often”. Answers 1 and 2 were scored 0 and answers 3 and 4 were scored 1 with a total of 28 points. A total score of 6 or higher was considered representative of a mental health problem.

The self-care behaviors questionnaire was adapted to be suitable for elderly people from previous research (18, 22, 23). It consisted of 24 items about mask-wearing behavior, hand-washing behavior, and social distancing behavior. The items were rated as follows: “never” (0 times/week), “sometimes” (1–4 times/week), and “regularly” (≥5 times/week). The total score was used to define the three categories: “high scores” (≥80% or ≥ 57 points), “moderate scores” (60%−79% or 44–56 points), and “low scores” (< 60% or ≤ 43 points).

The short version of the Quality of Life Assessment Scale developed by the World Health Organization translated to Thai (24, 25) was used. It consists of 26 items corresponding to four domains: physical health (7 items), psychological health (6 items), social relationships (3 items), and environmental health (8 items). Each item can be responded in a 1–5 scale. The total scores defined the three following categories: “poor quality of life” (score: 26–60), “moderate quality of life” (score: 61–95), and “good quality of life” (score: 96–130).

All the questionnaires were tested for content validity by three experts in their respective fields (i.e., internal medicine, behavioral health, and elderly nursing). The questionnaires were technically validated using the item-objective congruence score, where questions with a score ≤ 0.5 are eliminated, questions with a score of 0.5–0.69 are revised based on the experts' comments and feedback, and questions with a score equal to or above 0.7 are considered acceptable for data collection. The questionnaires passed a preliminary check with a test sample (N = 30) with similar characteristics to the study samples and the study area. The reliability of all the questionnaires except for the socio-demographic information questionnaire was tested with Cronbach's alpha coefficient, resulting in 0.77, 0.73, 0.79, 0.90, and 0.86, respectively.

### Statistical analysis

Statistical analysis was performed using the SPSS software (SPSS Inc., Chicago, IL, USA). The general information was described using mean, standard deviation (SD), minimum (Min), maximum (Max), frequency, and percentage. The Pearson correlation coefficient (r) was used to examine the correlation between COVID-19 knowledge score and outcomes, including scores of stress, mental health, self-care behaviors, and quality of life among elderly with NCD. Multiple linear regression was used to investigate factors associated with stress, self-care behaviors, and quality of life. Multiple logistic regression was also used to determine the outcome of a mental health problem. The final multivariable model was presented, in which all factors were found to be significant at the 0.05 level.

## Results

As for the demographic information (Table 1), the majority of the participants were women (56.0%) with a mean age of 69.42 (SD = 7.06). More than half of the sample were married (59.3%), educated (65.3%), unemployed (52.0%), and with sufficient income (60.7%). Regarding the health status data, almost all of them had never smoked (93.3%) and did not drink alcohol (78.7%). The sample had the following NCDs: hypertension (76.4%), diabetes mellitus (38.2%), dyslipidemia (43.1%), heart disease (4.9%), or other diseases such as COPD, tuberculosis, and gout (4.7%). As for comorbidities, almost half of them (47.8%) reported having one disease, two-thirds (37.1%) reported having two diseases, and less than one-fifth of the sample (15.1%) reported suffering from more than three diseases. The average treatment duration was 8.34 years (SD = 0.25). During the COVID-19 pandemic, about 13.6% of the participants visited a doctor at a hospital or according to the scheduled appointment. On the occasion of an illness, most of them (93.8%) reported visiting a doctor despite government measures. In terms of information about COVID-19, they reported receiving information from family members (76.9%), the television/radio (72.4%), health volunteers (70.9%), social media (41.3%), staff (36.4%), and posters (4.4%). Additionally, most of them (86.0%) had had access to an RT-PCR test for COVID-19.

**Table 1:** General characteristics of participants (*n* = 450).

**Variables**	***n* (%)**
**Sex**	
Male	198 (44. %)
Female	252 (56. %)
**Age (years)**	
60–69	250 (55.6%)
70–79	144 (32.0%)
≥80	56 (12.4%)
Mean ± SD	69.42 ± 7.06
Min.–Max.	60–97
**Marital status**	
Married	267 (59.3%)
Single/widowed/separated	183 (40.7%)
**Education**	
No	156 (34.7%)
Yes	294 (65.3%)
**Occupation**	
No	234 (52.0%)
Yes	216 (48.0%)
**Financial status**	
Insufficient	177 (39.3%)
Sufficient	273 (60.7%)
**Smoking**	
No	420 (93.3%)
Yes	30 (6.7%)
**Drinking alcohol**	
No	354 (78.7 %)
Yes	96 (21.3%)
**Complication**	
1 disease	215 (47.8%)
2 diseases	167 (37.1%)
3 diseases	68 (15.1%)
**Type of disease**	
Hypertension	344 (76.4%)
Diabetes mellitus	172 (38.2%)
Dyslipidemia	194 (43.1%)
Heart disease	22 (4.9%)
Other (COPD, Tuberculosis, Gout)	21 (4.7%)
**Duration of received treatment**	
0–5	183 (40.7%)
6–10	183 (39.3%)
≥10	183 (20.0%)
Mean ± SD	8.34 ± 0.25
Min.–Max.	1–30
**Visiting a doctor by appointment**	
No	389 (86.4%)
Yes	61 (13.6%)
**Visiting a doctor in case of illness**	
No	28 (6.2%)
Yes	422 (93.8%)
**Getting COVID-19 information**	
Family	346 (76.9%)
Television/Radio	326 (72.4%)
Volunteer	319 (70.9%)
Social media	186 (41.3%)
Staff	164 (36.4%)
Poster/Placard	20 (4.4%)
**RT-PCR test for COVID-19**	
Ever with negative	240 (53.3%)
Ever with positive	138 (30.7%)
Never	72 (16.0%)
**COVID-19 knowledge**	
Low level (scores 0–7)	205 (45.6%)
Moderate level (scores 8–9)	157 (34.9%)
High level (scores 10–12)	88 (19.5%)
**Stress level**	
Mild level (scores 0–24)	75 (16.7%)
Moderate level (scores 25–42)	141 (31.3%)
High level (scores 43–62)	89 (19.8%)
Severe level (scores 63–100)	145 (32.2%)
**Self-care behaviors**	
Low level (scores 24–43)	292 (64.9%)
Moderate level (scores 44–56)	42 (9.3%)
High level (scores 57–72)	116 (25.8%)
**Mental health problem**	
No (scores < 6)	297 (66.0%)
Yes (scores ≥ 6)	153 (34.0%)
**Quality of life**	
Low level (scores 26–60)	98 (21.8%)
Moderate level (scores 61–95)	318 (70.7%)
High level (scores 96–130)	34 (7.5%)

The mean score of COVID-19-related knowledge was 7.52 (SD = 2.2). The participants had low and moderate knowledge scores (45.6, 34.9%), followed by high scores (19.5%). Two-thirds of the sample presented severe (32.2 %) or moderate (31.3%) stress, while less than one-fifth fell into the categories of high (19.8%) or low (16.7%) stress (Mean = 46.76; SD = 21.48). According to the Thai GHQ-28, 34% of the participants presented mental health problems (Mean = 4.54, SD = 5.02). Regarding self-care behaviors, 64.9% of the sample reported a low level of self-care behaviors, followed by a high (25.8%) or moderate (9.3%) level (Mean = 42.68, SD = 13.67). As for the quality of life, 70.7% had a moderate quality of life, followed by low (21.8%) and high (7.6%) quality of life (Mean = 76.92, SD = 14.86) (Tables 1, 2). The results of the Pearson correlation analysis are presented in Table 2. Statistically significant associations were found between the knowledge score and the scores corresponding to stress (*r* = −0.846, *p* < 0.001), mental health (*r* = −0.743, *p* < 0.001), self-care behaviors (*r* = 0.500, *p* < 0.001), and quality of life (*r* = 0.332, *p* < 0.001).

**Table 2:** Correlation between COVID-19 knowledge, stress, mental health, self-care behaviors, and quality of life among elderly patients with non-communicable diseases.

**Factor**	**Mean ±SD**	**Range**	**1**	**2**	**3**	**4**	**5**
1. COVID-19 knowledge (scores)	7.52 ± 2.20	1–12	1				
2. Stress (scores)	46.76 ± 21.48	3–96	−0.846**	1			
3. Mental health (scores)	4.54 ± 5.02	0–23	−0.743**	−0.849**	1		
4. Self-care behaviors (scores)	42.68 ± 13.67	27–71	0.500**	−0.650**	−0.581**	1	
5. Quality of life (scores)	76.92–14.86	32–112	0.332**	−0.391**	−0.419**	0.298**	1

**Significant at the 0.01 level (2-tailed).

Table 3 presents the variables duration of the received treatment, hypertension, RT-PCR test for COVID-19, visiting a doctor in case of illness, and COVID-19-related knowledge that were significantly associated with the stress scores (*R*^2^ = 73.4%, *p* < 0.05). Interestingly, gender, hypertension, dyslipidemia, heart diseases, duration of the received treatment, and COVID-19-related knowledge were significantly associated with self-care behaviors (*R*^2^ = 28.8%, *p* < 0.05). Moreover, diabetes mellitus, hypertension, dyslipidemia, heart disease, RT-PCR test for COVID-19, visiting a doctor by appointment, visiting a doctor in case of illness, and COVID-19-related knowledge were significantly correlated with quality of life (*R*^2^ = 31.4%, *p* < 0.05). The logistic regression analysis revealed that married status (OR = 2.30, 95% CI = 1.14–4.64), alcohol consumption (OR = 0.30, 95% CI = 0.11–0.81), duration of the received treatment every 5 years (OR = 0.55, 95% CI = 0.36–0.86), history of COVID-19 detection (OR = 4.48, 95% CI = 1.45–13.84), and COVID-19-related knowledge (OR = 0.23, 95% CI = 0.17–0.31) were significantly associated with mental health problem (*p* < 0.05) (Table 4).

**Table 3:** Association between the COVID-19 knowledge influencing stress, self-care behaviors, and quality of life among elderly with NCD by multiple linear regression.

**Outcome**	**Factor**	**B**	**S.E**.	**Beta**	***P*-value**	**95% CI**	**VIF**
Stress	Constant	113.284	3.60		<0.001	106.21, 120.35	
	Duration of received treatment (5-years)	−1.935	0.72	−0.068	0.007	−3.34, −0.52	1.051
	Hypertension (yes)	1.895	1.08	0.044	0.081	−0.24, 4.02	1.045
	RT-PCR test for COVID-19						
	Ever with negative	−0.344	1.30	−0.008	0.793	−2.91, 2.226	1.539
	Ever with positive	6.601	1.83	0.113	<0.001	3.00, 10.19	1.628
	Never	Ref.					
	Visiting a doctor in case of illness (yes)	−4.703	2.24	−0.053	0.037	−9.11, −0.29	1.065
	COVID-19 Knowledge (score)	−8.020	0.27	−0.823	<0.001	−8.55, −7.48	1.284
**Self-care behaviors**
	(Constant)	20.537	2.75		<0.001	15.14, 25.93	
	Gender (female)	−2.368	1.11	−0.086	0.034	−4.55, −0.18	1.017
	Hypertension (yes)	−3.007	1.34	−0.093	0.025	−5.64, −0.37	1.080
	Dyslipidemia (yes)	−3.389	1.13	−0.123	0.003	−5.61, −1.16	1.052
	Heart disease (yes)	−7.263	2.67	−0.115	0.007	−12.52, −2.00	1.111
	Duration of received treatment (5-years)	1.776	0.74	0.098	0.017	0.32, 3.23	1.034
	COVID-19 Knowledge (score)	3.243	0.25	0.523	<0.001	2.75, 3.73	1.026
Quality of life	(Constant)	73.561	4.10		<0.001	65.50, 81.61	1.317
	Diabetes mellitus (yes)	−4.293	1.38	−0.141	0.002	−7.01, −1.57	1.215
	Hypertension (yes)	−4.288	1.52	−0.123	0.005	−7.28, −1.30	1.130
	Dyslipidemia (yes)	−3.522	1.25	−0.118	0.005	−5.99, −1.05	1.157
	Heart disease (yes)	−5.830	2.92	−0.085	0.047	−11.58, −0.08	1.157
	RT-PCR test for COVID-19						
	Ever with negative	1.972	1.57	0.066	0.210	−1.12, 5.06	1.788
	Ever with positive	−12.639	2.16	−0.312	<0.001	−16.90, −8.37	1.840
	Never	Ref.					
	Visiting a doctor by appointment (yes)	4.285	1.85	0.099	0.021	0.63, 7.93	1.175
	Visiting a doctor in case of illness (yes)	4.204	2.51	0.068	0.095	−0.74, 9.14	1.075
	COVID-19 knowledge (score)	1.437	0.30	0.213	<0.001	0.85, 2.03	1.280

B, unstandardized coefficients; S.E., standard error; Beta, standardized coefficients; 95% CI, 95% confidence interval; VIF, variance inflation factor; Ref., reference group.

**Table 4:** Association between the COVID-19 knowledge and mental health problem by multiple logistic regression.

**Factor**	**B**	**S.E**.	***P*-value**	**OR**	**95% CI**
Constant	10.553	1.35	<0.001	38283.29	
Marital status (married)	0.833	0.35	0.020	2.30	1.14,4.64
Drinking alcohol (yes)	−1.197	0.50	0.018	0.30	0.11,0.81
Duration of received treatment (5-years)	−0.589	0.22	0.009	0.55	0.36,0.86
RT-PCR test for COVID-19			<0.001		
Ever with negative	−0.523	0.44	0.239	0.59	0.25,1.41
Ever with positive	1.500	0.57	0.009	4.48	1.45,13.84
Never	Ref.				
COVID-19 knowledge (score)	−1.480	0.15	<0.001	0.23	0.17,0.31

B, regression coefficient; S.E., standard error; OR, odds ratio; 95%CI, 95% confidence; Ref., reference group.

## Discussion

Our results showed that the majority of the sample had low to moderate COVID-19-related knowledge scores, confirming Bandura's (26) concept that individual learning affects behavioral change. Highly believing in one's abilities results in a better prediction of the outcome (26). Additionally, environmental factors play an important role in determining individual behaviors (23). Knowledge is associated with individual behaviors (27) and is essential to healthcare since its correct and useful application leads to self-awareness of health conditions (28). Moderate knowledge related to COVID-19 is associated with prevention behaviors (23). In the context of COVID-19 prevention, elderly people with low levels of health literacy living in rural areas presented a detrimental health status (28). Similar to the present study, it has been shown that elderly people with chronic diseases have a low level of knowledge regarding the spread, common symptoms, prevention, and control of COVID-19 (28). However, in China, elderly people with NCDs such as diabetes and hypertension had variable levels of knowledge, possibly due to socio-demographic factors (29, 30).

In terms of stress, we found that more than half of the participants had moderate to severe stress. One possible explanation is the experience of three pandemic waves in the area with strict government measures against COVID-19 and suspension of traveling. Consistent with our findings, during the COVID-19 pandemic, stress and anxiety levels increased, especially among NCD patients (1, 31). The COVID-19 pandemic has disrupted almost every aspect of life and presents a unique threat to the physical and emotional wellbeing of the elderly, with a major portion of them having experienced moderate or severe health effects due to COVID-19 (32). According to the 2019 Thai Mental Health Data Report, where the vast majority of Thai people reported problems originating in stressful situations, age correlated with stress and anxiety (33). In line with this, it has been shown that elderly people experience stress that affects their daily life, with subsequent negative impacts on their physical and psychological health (34).

COVID-19 has resulted in severe mental issues, especially among the elderly with chronic illnesses (35). Here, one-third of the participants presented mental health problems as evaluated with GHQ-28 (scores ≥ 6 in 34% of the sample). This is probably due to the period of the assessment, which coincided with the third wave of COVID-19. With aging, physical functions decline, causing a higher risk of NCDs among older age groups. During the first lockdown, NCD patients in Europe and the United States experienced mental health problems (36). COVID-19 survivors and 60% of the controls presented pathologies as evaluated with GHQ-28 and anxiety as evaluated with the GAS-10 scale (37). In a recent study (38), nearly 40% of the elderly participants reported having mental health problems as a result of the COVID-19 pandemic. In another report (39), more than half of the participants (52.1%) felt fear and anxiety due to the COVID-19 outbreak. The pandemic caused long- and short-term psychological distress in the elderly population, potentially affecting their quality of life (40).

COVID-19 has become an ongoing crisis, significantly affecting the quality of life of all populations (41). Most of the participants (70.7%) obtained moderate scores in the quality of life assessment. Quality of life is negatively correlated with stress and mental health. A recent systematic review (42) indicated that quality of life deteriorated during the COVID-19 outbreak, especially among elderly people with NCDs. In another study, the majority of the participants had moderate scores of quality of life due to a decline in physical functioning and limitations concerning daily activities (43). In Turkey, at-risk populations had poorer quality of life during the pandemic, depending on their demographic background, medical condition, and psychological factors (44). Here, COVID-19-related knowledge, stress, mental health, and healthcare behaviors were associated with quality of life in elderly patients with NCDs.

The pandemic, being a global public health emergency, severely impacted the healthcare system worldwide (1), a situation that inevitably affects physical and psychological health, especially among elderly people (45). In the present study, time until receiving treatment, NCDs, RT-PCR tests for COVID-19, receiving medical services (“When you have symptoms, you can go to the doctor immediately”), and COVID-19-related knowledge were associated with stress. Moreover, this was a predictor of COVID-19-induced stress. Elderly people with underlying medical conditions experienced high levels of stress after the COVID-19 outbreak. Due to government-issued restrictions on traveling, limitation of the number of patients per day receiving health services, and social distancing, elderly people with NCDs did not receive health and medical services in time (9). Postponing such services led to the worsening of their chronic health conditions. The pandemic has hindered access to medical services and public transportation, especially for the elderly (46). Some reports described emergency cases of chronic NCD patients that did not receive the necessary health services (46, 47). Many patients report facing difficulties to access routine medical care and receive medication or health screenings, situations that cause anxiety and stress about their health condition (31).

The variables of gender, hypertension, dyslipidemia, heart disease, time until receiving treatment, and COVID-19-related knowledge significantly correlated with self-care behaviors. Importantly, these variables were predictors of self-care behaviors during COVID-19 for ~28% of the elderly with congenital diseases. In our study, most of the participants had low levels of self-care behaviors. This might be due to their age, when self-care behaviors are diminished, especially among chronic NCD patients, since underlying diseases affect daily life functionality (43). This is consistent with the concept that self-care behaviors arise from a learning process obtained from many components such as genetics, health status, the social environment, and individual experiences that influence self-care performance. An individual can achieve better health behaviors when relevant goals are set (26). In this context, a study with Indian populations found that moderate knowledge about COVID-19 resulted in relatively poor prophylactic behaviors (48). Additionally, the knowledge level has been linked to healthcare behaviors in hypertensive patients (49). Consistently, a systematic review suggested that knowledge affects self-care behaviors among elderly people (50). Therefore, programs that promote health-related behaviors can succeed if they include knowledge and prevention of risk factors (50).

Most of the inevitable health problems of chronic NCDs, for example, cancer, high blood pressure, osteoporosis, and diabetes, lead to reduced quality of life (51). It has been found that hypertension, dyslipidemia, and heart disease negatively correlate with self-care behaviors (52). In our study, diabetes mellitus, hypertension, dyslipidemia, heart diseases, RT-PCR tests for COVID-19, the statements “visiting a doctor to receive health services after an appointment” or “when you are sick, you immediately visit the doctor”, and COVID-19-related knowledge correlated with quality of life with a coefficient of multiple determination equal to 73.561. Furthermore, these variables were predictors of quality of life during COVID-19 (31.4%). Our findings are consistent with previous studies, where elderly with comorbidities and moderate depression experienced health-related deterioration of quality of life during the COVID-19 pandemic (14), especially in places where telemedicine was not accessible (53). The presence of underlying diseases was a major factor associated with quality of life. Moreover, congenital diseases, such as high blood pressure and diabetes, worsen elderly populations' lives (49), since health problems are linked to low quality of life in old age (53).

We found that alcohol consumption, time until receiving treatment, RT-PCR tests for COVID-19, and COVID-19-related knowledge significantly correlated with mental health. The knowledge scores were negatively associated with mental health. A person with a low level of knowledge may experience mental health disorders. Certain situations affect the commitment to practice healthcare behaviors. Thoughts and emotions (cognitive behaviors) can be adjusted to the environment that drives the motivation to perform such behaviors, depending on individual knowledge and experience (54, 55). Personal factors influence individuals' behaviors (55). It was documented that during the COVID-19 outbreak, anxiety in the elderly populations originated in pessimistic attitudes concerning mental health (56). Increased smoking and alcohol consumption during COVID-19 has been documented (57, 58).

This study has several limitations. First, the findings of this study were obtained from a cross-sectional design. Therefore, official mental health screening tests are necessary. In this context, the diagnosis of specific mental health disorders cannot be conducted through the information obtained from the questionnaires, since this requires a medical examination. Second, the third wave of COVID-19 in the area caused an increase in the number of infections, resulting in high scores of stress and mental health issues. The conclusions related to these variables should be considered with caution, taking into account the dynamics of COVID-19 and its consequences. Finally, the sample composition (elderly people with chronic NCDs, living in rural areas of northern Thailand) is very specific, and long-term studies are needed to confirm our results. In the future, research should focus on the needs of the elderly with chronic NCDs at the individual and community levels to provide solutions for the prevention and rehabilitation of mental health problems appropriate for the rural context of northern Thailand.

## Conclusion

The findings of this study suggest that the COVID-19 pandemic has had detrimental effects on elderly people with chronic NCDs. COVID-19-related knowledge positively correlated with healthcare behaviors and quality of life. Interestingly, several personal factors and COVID-19-related knowledge were associated with mental health during the third wave of the pandemic in rural areas of northern Thailand. This population faces multiple challenges concerning their physical and psychological health, as well as financial problems. Therefore, educational programs focusing on the severity of the disease, health complications, infectious diseases, and other emergencies are indispensable to improve health awareness and appropriate healthcare behaviors. Additionally, mental health support, stress management, accessible scheduled check-ups, health services, and medication are essential to the treatment of elderly people with NCDs and should be considered in relevant educational programs. Community stakeholders and policymakers should collaborate to address barriers and challenges to provide a timely, high-quality mental health service system to the aging population living in rural contexts and in accordance with their social culture.

## Data availability statement

The raw data supporting the conclusions of this article will be made available by the authors, without undue reservation.

## Ethics statement

The studies involving human participants were reviewed and approved by University of Phayao Human Ethics Committee, Thailand (UP-HEC-1.3/057/64). The patients/participants provided their written informed consent to participate in this study.

## Author contributions

All authors made substantial contributions to conception and design, data analysis, interpretation, involved in data collection, drafting the manuscript, revising it critically for important intellectual content, and given final approval of the version to be published. Each author should have participated sufficiently in the work to take public responsibility for appropriate portions of the content and agreed to be accountable for all aspects of the work in ensuring that questions related to the accuracy or integrity of any part of the work are appropriately investigated and resolved. All authors contributed to the article and approved the submitted version.

## Funding

This research was supported by School of Medicine (MD65-01) and the Thailand Science Research and Innovation Fund and the University of Phayao the Unit of Excellence.

## Conflict of interest

The authors declare that the research was conducted in the absence of any commercial or financial relationships that could be construed as a potential conflict of interest.

## Publisher's note

All claims expressed in this article are solely those of the authors and do not necessarily represent those of their affiliated organizations, or those of the publisher, the editors and the reviewers. Any product that may be evaluated in this article, or claim that may be made by its manufacturer, is not guaranteed or endorsed by the publisher.
